# Dopaminergic D2-like receptor stimulation affects attention on contextual information and modulates BOLD activation of extinction-related brain areas

**DOI:** 10.1038/s41598-023-47704-6

**Published:** 2023-11-28

**Authors:** Alina Nostadt, Michael A. Nitsche, Martin Tegenthoff, Silke Lissek

**Affiliations:** 1 Ruhr-University Bochum, Faculty of Psychology, 44789 Bochum, Germany; 2 Department of Psychology and Neurosciences, Leibniz Research Centre for Working Environment and Human Factors, Ardeystr. 67, 44139 Dortmund, Germany; 3 Department of Neurology, BG University Hospital Bergmannsheil, Ruhr-University Bochum, Bürkle de La Camp-Platz 1, 44789 Bochum, Germany; 4 German Centre for Mental Health (DZPG), 44789 Bochum, Germany; 5 University Hospital OWL, Protestant Hospital of Bethel Foundation, University Clinic of Psychiatry and Psychotherapy and University Clinic of Child and Adolescent Psychiatry and Psychotherapy, Bielefeld University, 33617 Bielefeld, Germany

**Keywords:** Extinction, Attention, Human behaviour

## Abstract

Contextual information is essential for learning and memory processes and plays a crucial role during the recall of extinction memory, and in the renewal effect, which is the context-dependent recovery of an extinguished response. The dopaminergic system is known to be involved in regulating attentional processes by shifting attention to novel and salient contextual cues. Higher dopamine levels are associated with a better recall of previously learned stimulus-outcome associations and enhanced encoding, as well as retrieval of contextual information which promotes renewal. In this fMRI study, we aimed to investigate the impact of processing contextual information and the influence of dopaminergic D2-like receptor activation on attention to contextual information during a predictive learning task as well as upon extinction learning, memory performance, and activity of extinction-related brain areas. A single oral dose of 1.25 mg bromocriptine or an identical-looking placebo was administered to the participants. We modified a predictive learning task that in previous studies reliably evoked a renewal effect, by increasing the complexity of contextual information. We analysed fixations and dwell on contextual cues by use of eye-tracking and correlated these with behavioural performance and BOLD activation of extinction-related brain areas. Our results indicate that the group with dopaminergic D2-like receptor stimulation had higher attention to task-relevant contextual information and greater/lower BOLD activation of brain regions associated with cognitive control during extinction learning and recall. Moreover, renewal responses were almost completely absent. Since this behavioural effect was observed for both treatment groups, we assume that this was due to the complexity of the altered task design.

## Introduction

In extinction learning, a conditioned response is no longer reinforced and as a result, it decreases. After successful extinction, an individual has learned that a previously learned stimulus-outcome association is no longer valid, thus the previous behavioural response ceases^1,2^. However, the extinguished response can recover, which suggests a failed recall from extinction memory^2^. One type of recovery is called renewal, which occurs when extinction learning is performed in a context that is different from acquisition and recall^2^*.* During a typical extinction learning paradigm, participants learn, extinguish, and retrieve stimulus-outcome associations during three different task phases: acquisition, extinction learning, and recall^3–7^. To test for a context-dependent recovery of an extinguished response (renewal effect), conditions with (ABA condition) and without context change (AAA condition) are compared. In previous studies of extinction learning, increasing salience and shifting attention toward contextual cues affected recall of extinguished stimulus-outcome associations, resulting in impaired recall of extinction memory and higher renewal rates^8^. It is known that attention and memory processes are closely linked to each other^9,10^. Attention allows individuals to acquire essential information about the environment, which can guide their behaviour and presumably aids during the recall of task-relevant information^9,10^. In associative learning, models of attention attempt to explain how humans attend to environmental stimuli for goal-directed behaviour^11–14^. The Mackintosh model of attention is known as the high-predictive model^11,15^. It claims that individuals shift their attention to environmental cues that provide the most relevant information to predict upcoming events^15^. In the context of conditioning theories, studies have shown that due to selective attention, the salience of a cue increased when it resulted in a smaller prediction error^15,16^. Pearce and Hall proposed a model stating that individuals would benefit from attending to cues that provide uncertain information and that the processing of such uncertain environmental cues is crucial for goal-directed behaviour^12^. This model is also supported by previous studies which showed that individuals can appropriately adjust their behaviour when they shift attention to and thus assign salience to uncertain cues^12,16^. Both models of attention have specific advantages for explaining goal-directed behaviour and extinction learning.

The dopaminergic system is known to influence learning processes and to modulate extinction learning^6,17^. It is functionally involved in the reward system, goal-directed behaviour, and attentional aspects of conditioning as it directs attention to salient and novel contextual cues^18,19^. Previous studies have shown that dopamine D2-like receptor stimulation affects activation in extinction-related brain areas such as the hippocampus (HC) and prefrontal regions such as the inferior frontal gyrus (iFG)^20–24^. The increase or decrease of dopaminergic levels affected the hippocampus-dependent memory^25,26^ and the activation of the inferior frontal gyrus (iFG) as well as the frontostriatal circuit while mediating response inhibition^27^. Also, D2-like receptor stimulation before extinction learning resulted in greater hippocampal activity and higher renewal levels^19^. Dopaminergic receptor availability was linked to higher iFG activation which was related to response inhibition^27,28^. On the contrary, other studies showed that a dopaminergic antagonist, which blocks dopamine receptors, impaired extinction and resulted in reduced hippocampal activation, without affecting renewal^6^. Also, dysfunction of the dopaminergic system is linked to impairments of attentional processes such as in attention deficits in hyperactivity disorders^29^.

Yet it is still largely unknown how attentional mechanisms and the processing of contextual information during extinction learning affect recall of stimulus-outcome associations. Furthermore, the effects of dopaminergic D2-like receptor stimulation on attention in the context of extinction learning and underlying neural mechanisms remain unclear. In our study, we investigated how the processing of contextual information affects extinction learning with and without dopaminergic D2-like receptor stimulation. In our predictive learning task, healthy human individuals learned, extinguished, and recalled stimulus-outcome associations. We used a modified version of a predictive learning task that was designed to reliably evoke a renewal effect in context-related extinction learning without a fear component^4,8,19,30–33^. Due to our focus on differences in processing contextual information and thus potentially varying effects upon extinction learning, we modified the contexts compared to earlier studies and presented a more realistic scenario with several salient contextual cues. As a measure of attention, we investigated participants’ context exploration during our predictive learning task: we recorded eye movement to investigate gaze behaviour and fixations of contextual information. In addition, we performed functional MRI to analyse underlying neural mechanisms. We mainly focused on brain regions that were previously found and associated with extinction learning, such as the hippocampus and left inferior frontal gyrus, and on regions that play a functional key role in the salience network, i.e., anterior cingulate cortex (ACC) and insula.

We hypothesized that salient contextual cues presented during all phases of our predictive learning task would increase overall renewal rates. Dopaminergic stimulation was assumed to strengthen the effect in terms of higher renewal rates compared to placebo treatment, due to higher attention to contextual cues. Based on previous findings^17,19,24,34,35^, we supposed that dopaminergic stimulation would promote the shift of attention toward salient contextual cues, due to dopaminergic effects upon hippocampal and prefrontal processing, supporting the recall of context-dependent extinction memory.

## Materials and Methods

### Participants

We recruited 50 volunteers to participate in our study on an online recruiting platform that specializes in the acquisition of potential study participants. Group sizes are based on a priori power analyses for the respective research question, calculated in fmripower^36,37^ and G*Power 3.1 for a power of 0.8 and an *α*- error of 0.05, based on effect sizes from previous studies^6,19^. We expected medium to large-sized effects of treatment and renewal propensity in behaviour and brain activation. All participants were enrolled as university students in various fields at the time of participation. We excluded psychology students from the third semester onwards since their potential familiarity with such tasks might influence their task performance. Standard exclusion criteria for MRI measurements were applied and we only included right-handed participants (Edinburgh Handedness Inventory), excluding volunteers with a history of neurological or mental disorders (self-reported), regular intake of medicine, a body mass index outside the range of 18–27 kg/m^2^, age outside the range of 18–35 years, drug use and smoking. Participants were randomly assigned to the experimental bromocriptine and placebo control group. During data analyses, we excluded n = 4 participants from the bromocriptine group and n = 1 from the placebo group due to excessive head movement or incomplete data sets. In total, the data sets of 45 volunteers (29 females, 23 males) mean age of 24.4 (± 4.6 SD; range 19–35) years were included in the analysis. The mean age within the bromocriptine group (n = 21, 14 women; range 19–34) was 24.8 (± 4.6 SD) years and 24.1 (± 4.6 SD) years in the placebo group (n = 24, 17 women; range 19–35).

### Ethics statement

Before the experiment, participants received detailed information about the fMRI procedure and pharmacological properties and potential side effects of the DA-agonist bromocriptine. All subjects participated in this study after giving written informed consent and received monetary compensation (50€). The protocol was approved by the Ethics Committee of the Ruhr-University Bochum (Registration No. 16-5738) and conforms to the Code of Ethics of the World Medical Association (Declaration of Helsinki).

### Experimental procedure

In this double-blind study, we applied a randomized design. Participants received either a single oral dose of 1.25 mg bromocriptine (MEDA Pharma GmbH & Co. KG) or an identical-looking placebo. The drugs were administered 90 min before the start of the predictive learning task, following the pharmacokinetic profile of bromocriptine with peak plasma concentrations achieved around this time point^38^. The participants performed all three task phases (acquisition, extinction, recall) in succession.

### Predictive learning task

**Figure 1:**
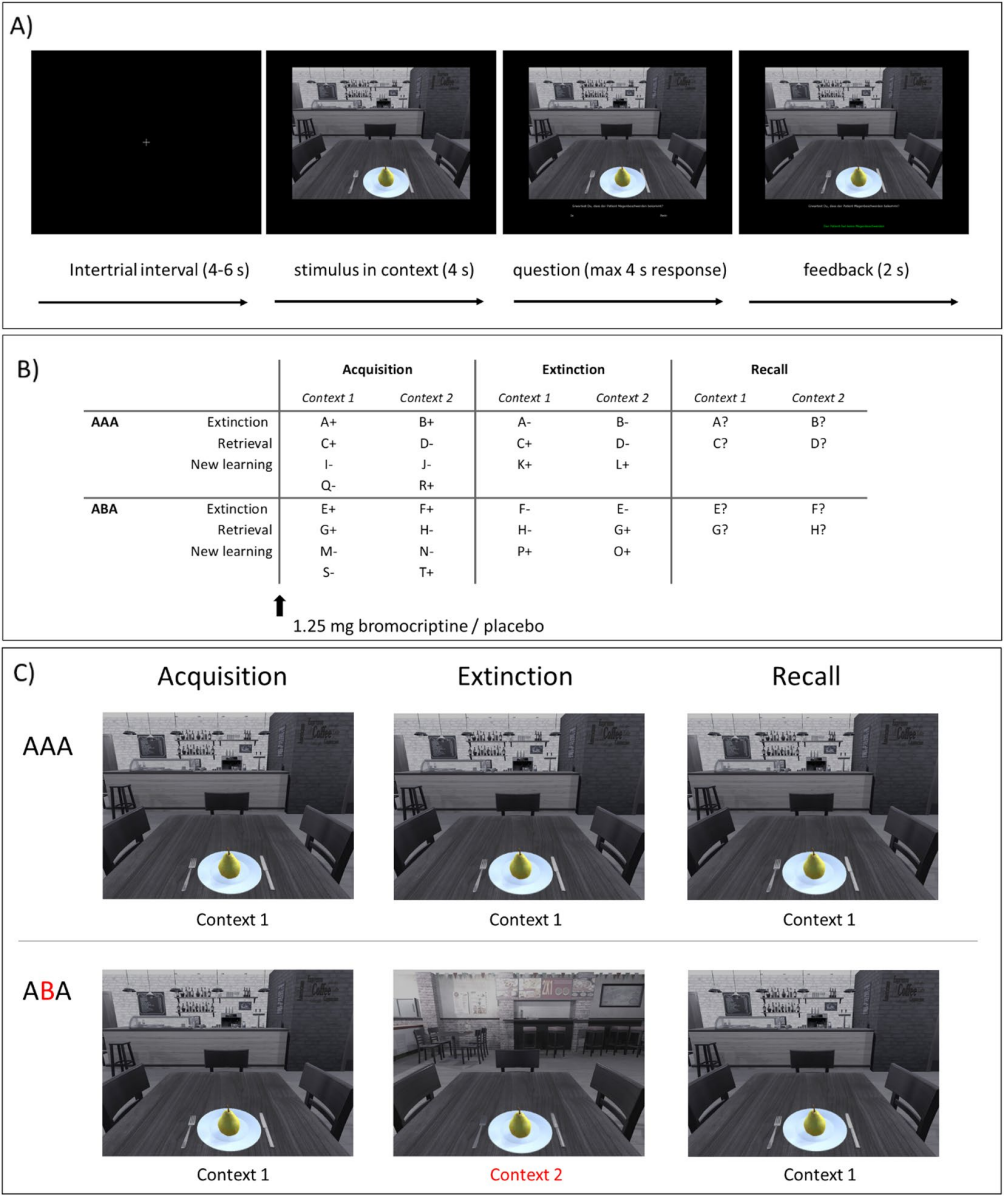
Predictive learning task. (**A**) Trial example. Participants learned to predict whether food that is served in a restaurant causes stomachache or not. The intertrial interval was 4–6 s, and at the beginning of each trial, the stimulus was presented in its context for 4 s. After the 4 s had elapsed, a question was displayed on the screen below the food stimulus (“Do you expect your patient to get a stomachache?”) for a maximum of 4 s response time. Feedback was shown for 2 s, providing the correct answer, “The patient does not have a stomachache.” or “The patient has a stomachache.” (**B**) Experimental design of the predictive learning task for extinction, retrieval, and new learning conditions. Letters represent the food stimulus, plus and minus indicate if the stimulus predicts stomachache or not (‘ + ’: stimulus predicts stomachache; ‘−‘: stimulus predicts no stomachache). In condition AAA during extinction, the stimulus occurs in the same context as during acquisition. In condition ABA during extinction, the stimulus occurs in a context different from that during acquisition. In both conditions, the final test (recall) for the renewal effect is performed in the context of acquisition. **(C)** Example of AAA and ABA trials. AAA and ABA indicate if there was a context change during the predictive learning task. In AAA trials (no context change), the food stimulus was presented in one context during the acquisition, extinction, and recall phases. In ABA trials (context change), a stimulus was presented in a different context during the extinction learning phase.

The predictive learning task used in this study is a modified version of a learning task that was designed to reliably evoke a renewal effect in context-related extinction learning without a fear component^30^. Previous studies already used this task in a different version adapted for fMRI experiments^4,5,19,32,39^. Participants were asked to put themselves in the position of a physician and predict whether various food items (fruit or vegetable) served in two different restaurants would lead to the aversive consequence of a stomachache in their patient. We presented the food item in the centre of the screen. In the background, we displayed one of two restaurant images that contained different contextual cues in the upper third of the screen. At the beginning of each trial, the stimulus in its context was first presented for 4 s. After the 4 s had elapsed, a question asking whether the patient will develop stomachache was shown, together with the response options “Yes” or “No” for maximum 4 s. Thus, participants had a maximum response time of 4 s and responded by pressing the respective button with the right hand. After the response, or after the expiration of the response time, feedback with the correct answer was displayed for 2 s underneath the food stimulus (“The patient has a stomachache” or “The patient does not have a stomachache”) (see Fig. 1). Our task consisted of two learning phases (acquisition and extinction learning) and the recall (a test phase).

*Acquisition* Participants learned to associate a food item that was presented either in context A or B, with its consequence. The food stimuli were presented in randomized order. The acquisition phase contained 16 different food stimuli, 8 stimuli per context. Each stimulus was presented eight times, amounting to a total of 128 trials. Half of the stimuli predicted stomachache and the others predicted no stomachache. The consequence of stomachache was counterbalanced to appear equally often in both contexts.

*Extinction learning phase* Some of the previously learned stimulus-outcome associations were extinguished (extinction stimuli) while others retained their consequence (retrieval stimuli). In addition, some stimuli were presented in a different context (AAA or ABA conditions indicate if there was a context change) during the predictive learning task (see Fig. 1B for a detailed overview of AAA/ABA and extinction and retrieval conditions).

In the extinction learning phase of our predictive learning task, eight (half of the stimuli from the acquisition phase) were presented again. Half of these eight (four) were presented again in context A as during acquisition (AAA—condition *without context change*) and the other half (four) in a different context B (ABA—condition *with context change*) in randomized order. Within these groups of stimuli, a further distinction was made between actual *extinction* stimuli (i.e., stimuli for which the consequence of stomachache changes and *retrieval* stimuli (for which the consequence of stomachache does not change), resulting in each two extinction stimuli and two retrieval stimuli per context. This design results in four different experimental conditions during the extinction learning phase: AAA Extinction (consequence change & no context change), AAA Retrieval (no consequence change & no context change), ABA Extinction (context change & consequence change), and ABA** Retrieval** (context change & no consequence change). In addition, four new stimuli were introduced during the extinction phase, to balance the design to contain equal numbers of stimuli predicting stomachache in both contexts (new learning). Therefore, the extinction phase contained a total of 12 different stimuli, 6 per context, with each stimulus being presented eight times, amounting to a total of 96 trials. Half of the stimuli predicted stomachache, the other half predicted no stomachache, and the consequence of stomachache was counterbalanced to appear equally often in both contexts. In all other respects, trial design was identical to acquisition.

*Recall (test phase)* This phase consisted of 40 trials in total, in which extinction and retrieval stimuli were presented again. Half of the extinction and retrieval stimuli were presented again in the context of acquisition, the other half were presented in the same context as during extinction. During this phase, we tested the memory performance and especially the context-dependent extinction memory of the participants. When participants responded to ABA extinction stimuli as they had learned during acquisition, they showed context-dependent renewal. For further explanation of our data analysis, see section "Behavioural data analysis". With the exception that participants received no feedback at all during the recall phase, trials were identical to those during acquisition. See Fig. 1 for an overview of the task design.

### Data acquisition

We applied the same protocol that we have used and described in previous fMRI studies. Functional and structural brain scans were acquired using a whole-body 3T scanner (Philips Achieva 3.0 T X-Series, Philips, The Netherlands) with a 32-channel SENSE head coil. Blood-oxygen-level-dependent (BOLD) contrast images were obtained with a dynamic T2* weighted gradient echo EPI sequence using SENSE (TR 3200 ms, TE 35 ms, flip angle 90°, field of view 224 mm, slice thickness 3.0 mm, voxel size 2.0 × 2.0 × 3.0 mm)^7,8^. We acquired 45 transaxial slices parallel to the anterior commissure—posterior commissure (AC-PC) line which covered the whole brain^8,32^. High-resolution structural brain scans of each participant were acquired using an isotropic T1 TFE sequence (field of view 240 mm, slice thickness 1.0 mm, voxel size 1 × 1 × 1 mm) with 220 transversally oriented slices covering the whole brain^32^. The task was presented to the participants via an fMRI-ready monitor (BOLDscreen 24 LCD, Cambridge Research Systems) connected to a computer that ran specific software programmed in MATLAB (V.2022b, The Math Works, USA). Responses were given using an fMRI-ready keyboard (Lumitouch response pad, Photon Control Inc., Canada)^8,32^. Eye movement and gaze behaviour were recorded with a sampling rate of 500Hz for one eye using the Eye-Tracking system EyeLink 1000 Plus (SR Research Ltd., Canada) of each participant while they performed all learning phases of our predictive learning task.

### Eye-Tracking

**Figure 2:**
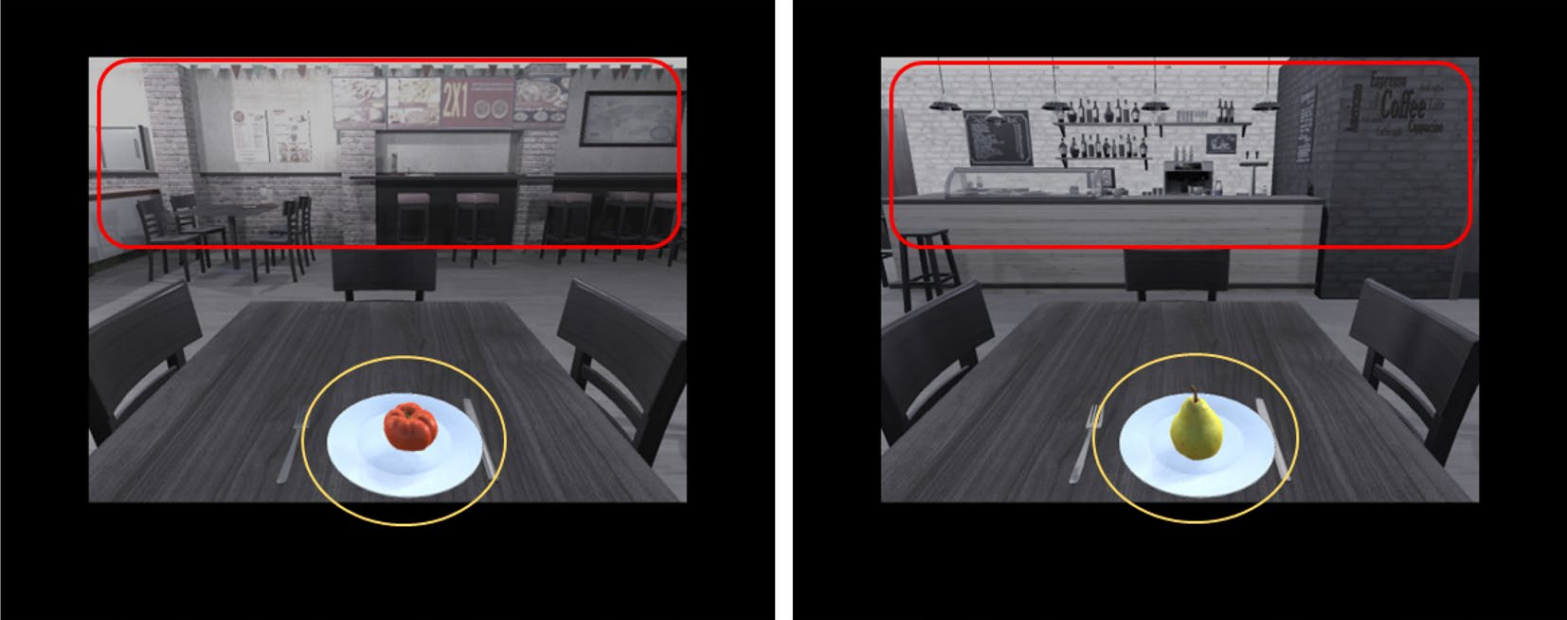
Example of how we defined ROIs (contextual cues and the food stimulus) for our eye-tracking analysis during the period of interest (stimulus onset-question onset). Red frames indicate contextual cues while yellow circles refer to the food stimulus. Context 1 and context 2 differ in features in the upper third of the pictures.

For analysis (EyeLink Data Viewer V.4.1.211, SR Research Ltd., Canada), we defined interest periods and ROIs of each task phase based on our experimental design for all participants. The interest period was defined as the interval between onsets of stimulus and question. During this time, the food stimulus was presented in one of two contexts and the participants could explore the contextual cues before a response was required (4 s after stimulus onset). As ROIs, we defined the food stimuli (presented in the middle of the screen) and context stimuli (salient contextual cues in the upper third of the screen) (see Fig. 2). For each participant, the number of fixations (mean fixation) and the fixation duration (mean dwell, ms) for food and context stimuli were calculated separately for learning phases and experimental conditions. To test if there are differences in eye movement behaviour between treatment groups and for experimental conditions (e.g., with and without context change), we statistically tested for differences as described in section "Results".

### Behavioural data analysis

For all three task phases, log files were recorded that contained information on stimulus type, context type, and correctness of response of each trial, from which we calculated error rates and memory performance for the different learning phases and experimental conditions. Errors in acquisition and extinction learning were defined as responses stating the incorrect association between the context-cue-compound and the consequence. During recall, we tested the correctness of response for retrieval stimuli (in both, context change and no context change) as well as the extinction memory. For extinction stimuli, we were interested in the context-dependent recall of extinction memory (i.e., the context-dependent renewal effect) and the recall of AAA extinction memory in the experimental condition without context change. In case of renewal, associations learned during acquisition in context A will reappear in the recall phase, which is again performed in context A, while extinction was performed in context B. In contrast, the AAA condition constitutes a control condition for extinction learning, since here all learning phases are performed in an identical context. A response that referred to the association which was correct during acquisition constituted an error in the AAA condition and a renewal response in the ABA condition. If extinction learning is successful, responses during the recall phase will reflect the associations learned during extinction. We calculated the mean and standard deviation of each participant's learning errors in each learning phase and experimental condition^8,32^. Our behavioural data analysis (e.g., calculation of learning errors and the renewal effect) was based on our previous research studies using the same predictive learning task^8,32^.

For a behavioural analysis on a group level, we used a Kolmogorov–Smirnov test to analyse the normal distribution of the data. Learning errors, fixations, and dwell were not normally distributed (*p* < 0.001), therefore we calculated median and interquartile range (IQR) to describe our data at the group level and non-parametric tests such as Wilcoxon signed-rank test and Spearman's rank correlation coefficient for statistical testing. Statistical analyses were performed using MATLAB (V.2022b, The Math Works, USA).

### Imaging data analysis

For preprocessing and statistical analysis of fMRI data, we used the software Statistical Parametric Mapping (SPM), Version 12 (Wellcome Department of Cognitive Neurology, London, United Kingdom), implemented in MATLAB (V.2022b, The Math Works, USA). Our imaging data analysis (e.g., preprocessing and second-level analysis) was based on our previous fMRI studies investigating the renewal effect of extinction learning^6,8,32^. Three dummy scans, during which the BOLD signal reached a steady state, preceded the actual data acquisition of each session, thus preprocessing started with the first acquired volume. Preprocessing on a single subject level consisted of the following steps: slice-timing correction to account for time differences during image acquisition; realignment of all volumes to the first volume for motion correction; spatial normalization into standard stereotactic coordinates with 2 × 2 × 2 mm^3^ using an EPI template of the Montreal Neurological Institute (MNI) provided by SPM, smoothing with a 6 mm full-width half-maximum (FWHM) kernel, following the standard SPM procedure^32^. The acceptable limit for head motion was 3 mm for translational movements and 1.5° for rotational movements. If these limits were exceeded in a single volume or across the whole scanning session, the data of the respective participants were excluded from further analysis^32^.

In a first-level analysis, we calculated BOLD activity during the acquisition, extinction, and recall phases. For the extinction learning and recall phase, activation was additionally calculated for the respective experimental conditions, i.e., extinction/retrieval and context change/no context change. We modelled regressors for the onset of each context-cue compound. All regressors were modelled using distinct stick functions convolved with the canonical hemodynamic response function in the general linear model implemented in SPM, in an event-related design (duration = 0). We calculated individual contrasts based on the onset of the image of the context-cue compound at the beginning of a trial, compared to the baseline. The beta values that we extracted during the first-level analysis, were entered the contrast images in a second-level random-effects analysis. The activation patterns of all participants were calculated in a one-sample t-test. Here, we inserted learning errors, fixations, and dwell on contextual cues, as covariates of interest (COI) together in one general linear model. For each task condition, one-sample t-tests were calculated separately. To compare the BOLD activation of both treatment groups (bromocriptine/placebo), the contrast images from the single subject were entered into a flexible factorial design containing the between-subjects factor treatment (bromocriptine/placebo) and the within-subjects factor task phase (i.e., calculated contrast images for different task phases) and within-subjects factor experimental conditions, i.e., ABA (context change) and AAA (no context change) trials. In the two-sample t-test, we also added COI in the SPM flexible factorial design (learning errors, fixation, or dwell of contextual cues) for each task phase.

We used a whole brain, and region of interest (ROI) analyses based on our hypotheses. Our a priori ROIs included brain regions (e.g., hippocampus, inferior frontal gyrus, prefrontal cortex, insula, medial temporal lobe), that previous studies had demonstrated to significantly contribute to extinction learning by processing context features, response selection/inhibition, and decision making^5,8,19,40,41^. For these regions we constructed anatomical ROIs consisting of the corresponding anatomical regions defined in the WFU pickAtlas Toolbox implemented in SPM 12, using AAL atlas regions^42^. In general, imaging results are reported in terms of significance on the whole-brain level with FWE correction, thresholded at *p* < 0.05 peak level. For results marked with an asterisk (^∗^), small volume correction was applied with FWE correction, thresholded at *p* < 0.05 peak level. In these cases, the respective small volume always consisted of the complete anatomical ROI.

## Results

### Fixation and dwell time on contextual cues and food stimuli

**Figure 3:**
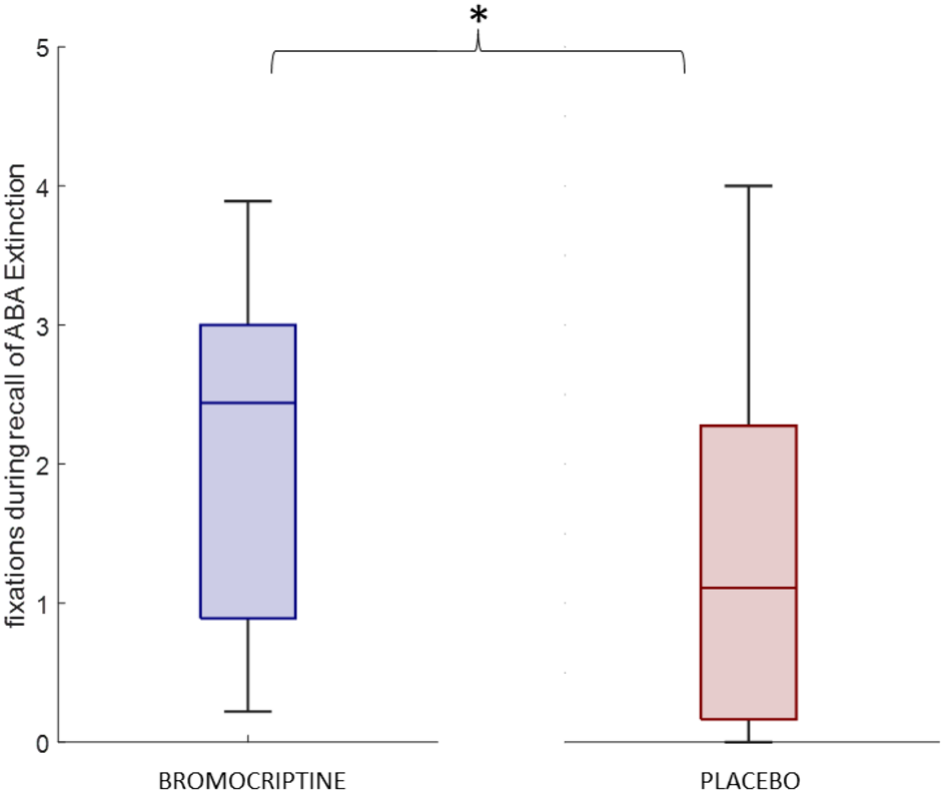
Fixations on contextual cues during recall of ABA extinction memory. The bromocriptine group had significantly more fixations on contextual cues during the recall of extinction memory in the condition with context change (ABA) compared to placebo.

In our analysis of eye movement, we observed differences in fixations and dwell of different stimulus types using a three-way ANOVA. We revealed a main effect of stimulus type on fixations [F(1) = 258.01, *p* < 0.001] and dwell [F(1) = 484.8, *p* < 0.001]. Treatment [fixation: F(1) = 0.48, *p* = 0.49; dwell: F(1) = 0.12, *p* = 0.73] and learning phase [fixation: F(2) = 0.66, p 0 51; dwell: F(2) = 0.1, *p* = 0.91] did not have a significant main effect nor an interaction effect (treatment*stimulus, treatment*learning phase, stimulus*learning phase; *p* > 0.5). In all three learning phases, participants had significantly more fixations and longer dwell on food stimuli compared to contextual cues (see Table 1). Food vs. Context (Acquisition: FIXATIONS Z = 7.15, *p* < 0.001, DWELL Z = 7.28, *p* < 0.001; Extinction: FIXATIONS Z = 7.79, *p* < 0.001, DWELL Z = 7.88, *p* < 0.001; Recall: FIXATIONS Z = 6.62, *p* < 0.001, DWELL Z = 6.57, *p* < 0.001). To analyse differences and underlying neural mechanisms for processing contextual information and their effects on extinction learning and recall of extinction memory, we continued our analysis with fixations and dwell on contextual cues. Based on our assumption that the bromocriptine group directs attention to novel and salient stimuli, we calculated a planned contrast (Wilcoxon signed-rank test, one-tailed) of both treatment groups in the ABA extinction condition (Z = 1.17, *p* = 0.04) (see Fig. 3). Results showed that the bromocriptine group had significantly more fixations on contextual cues during the recall of extinction memory in the ABA condition. Besides that, fixations and dwell on contextual cues were similar for all task phases and experimental conditions (see Table 2 for an overview).

**Table 1:** Median (IQR) of fixations and dwell (ms) on food stimuli and contextual cues during our interest period (between stimulus onset and question onset).

FIXATION		DWELL		
Food	Context	Food	Context	
5.72 (2.09)	1.62 (1.09)	2190.6 (1060.3)	387.07 (428.68)	Acquisition
6.31 (1.87)	1.31 (1.67)	2088.1 (866.96)	313.76 (434.8)	Extinction learning phase
6.22 (3.13)	1.77 (1.83)	2177.3 (774.73)	304.02 (441.73)	Recall phase

**Table 2:** Median (IQR) of fixation and dwell (ms) of contextual cues for all three task phases. Median of fixation and dwell of the four experimental conditions (extinction/retrieval and with/without context change) are shown for the extinction learning and recall phase.

CONTEXT				
	Acquisition			
Fixations	1.71 (1.09)			
Dwell (ms)	397.07 (428.67)			

	Extinction learning phase			
	*AAA (no context change)*		*ABA (context change)*	
	*Extinction*	*Retrieval*	*Extinction*	*Retrieval*
Fixations	1.2 (2.03)	1.47 (1.53)	1.53 (1.45)	1.33 (1.51)
Dwell (ms)	337.60 (484.02)	312.47 (443.83)	391.87 (406.6)	327.33 (451.38)

	Recall phase			
	*AAA (no context change)*		*ABA (context change)*	
	*Extinction*	*Retrieval*	*Extinction*	*Retrieval*
Fixations	1.56 (1.89)	1.44 (1.57)	1.44 (2.31)	2.0 (1.57)
Dwell (ms)	340.44 (448.91)	308.33 (563.99)	386.11 (485.86)	440.44 (516.94)

### Learning and memory performance

**Figure 4:**
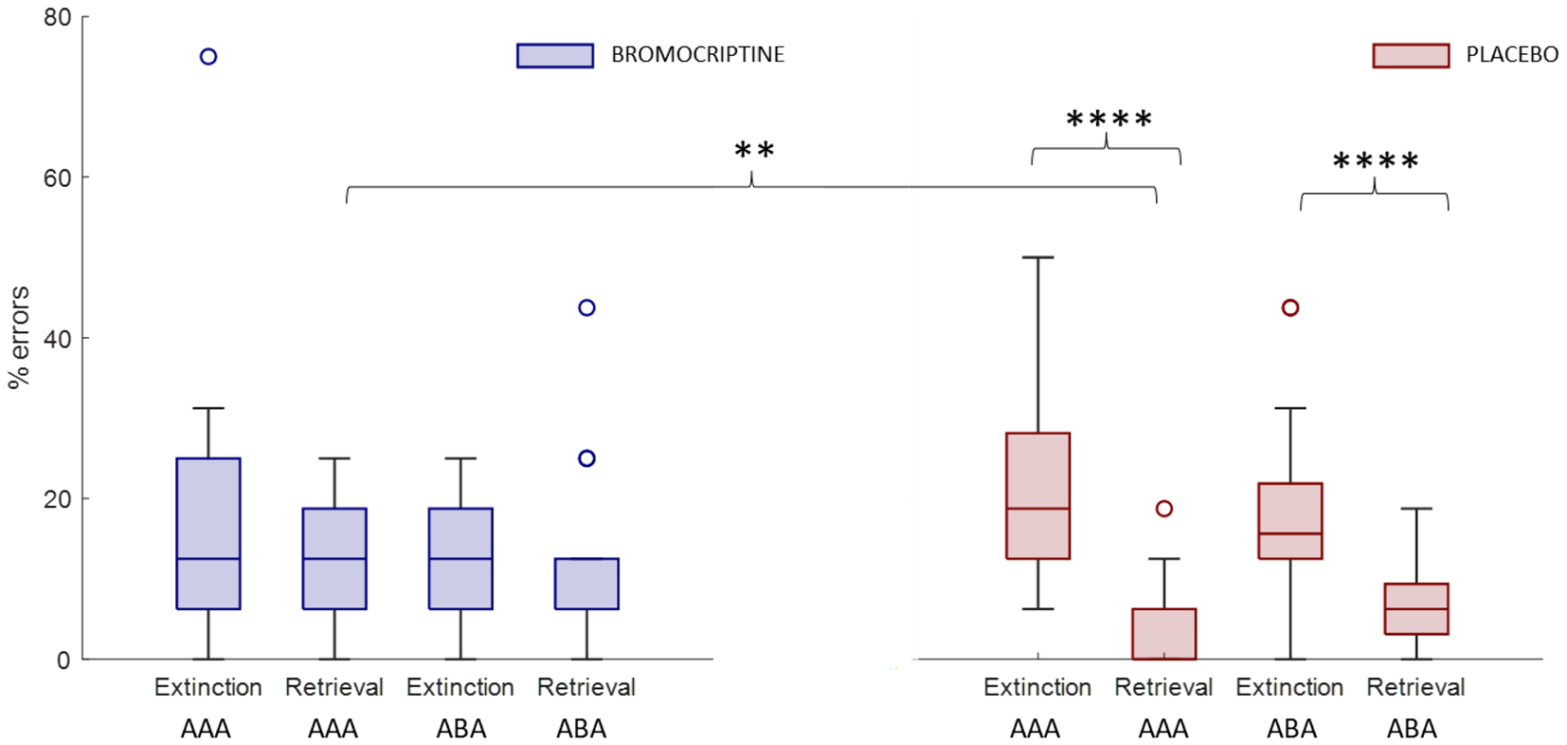
Overview extinction learning phase errors. On a group level, the median (IQR) was calculated for both treatment groups in extinction and retrieval conditions with and without context change. The bromocriptine group had similar learning errors in these experimental conditions, while the placebo group showed significant differences. Here, we observed significantly fewer learning errors in retrieval conditions compared to extinction in both, ABA (context change) and AAA (no context change) conditions. There was also a significant difference between treatment groups within retrieval conditions, with BRC showing a higher percentage of learning errors.

Learning performance, in terms of percent errors (median (IQR)), was equally good for both treatment groups (bromocriptine 13.28 (10.35), placebo 13.28 (9.76); Z = − 0.07, *p* = 0.9) during acquisition (Wilcoxon signed-rank test). We analysed the learning performance of all four experimental conditions during the extinction learning phase (ANOVA), finding a main effect of context change [F(1) = 32.87, *p* < 0.001] and an interaction effect (treatment*context change) [F(1) = 5.4, *p* = 0.02] on learning errors (no main effect of treatment [F(1) = 0.75 *p* > 0.3] and consequence change [F(1) = 0.75 *p* > 0.6]). Post hoc tests (Wilcoxon signed-rank test) showed that all participants had significantly more learning errors in extinction compared to retrieval in both conditions, with (Z = 4.37, *p* < 0.001) and without (Z = 4.41, *p* < 0.001) context change (see Table 3). A comparison between treatment groups revealed that the bromocriptine group’s learning performance was similar in all experimental conditions, while the placebo group had significantly lower error rates in the retrieval conditions without context change (AAA) compared to bromocriptine (AAA Retrieval: Z = 2.96, *p* = 0.003). For placebo, the learning performance was also different for extinction and retrieval conditions (AAA: Z = 5.15, *p* > 0.001; ABA: Z = 4.05, *p* > 0.001) (see Fig. 4). During recall, we observed no difference in recall performance in all four experimental conditions and between treatment groups. All participants had a similarly good recall of extinction and retrieval memory (see Table 3).

**Table 3:** Median (IQR) percent learning errors of all participants for the three task phases. Errors in four experimental conditions (extinction/retrieval and with/without context change) are shown for the extinction learning and recall phasae. ABA Extinction trials in the recall phase reflect the condition in which the renewal effect is evoked.

Acquisition			
13.28 (10.93)			

Extinction learning phase			
*AAA (no context change)*		*ABA (context change)*	
*Extinction*	*Retrieval*	*Extinction*	*Retrieval*
18.75 (12.5)*	6.25 (12.5)*	12.5 (6.25)**	6.25 (6.25)**

Recall phase			
*AAA (no context change)*		*ABA (context change)*	
*Extinction*	*Retrieval*	*Extinction*	*Retrieval*
0 (0)	0 (0)	0 (0)	0 (0)

*Z = 4.41, *p* < 0.001.

**Z = 4.37, *p* < 0.001.

### Imaging results

To analyse to what extent activation patterns of extinction-related brain areas are influenced by shifting attention to contextual cues, we introduced fixations and dwell on contextual cues as COI. Moreover, we compared BOLD activation between treatment groups using a two-sample t-test to reveal possible effects of the dopaminergic receptor stimulation.

**Figure 5:**
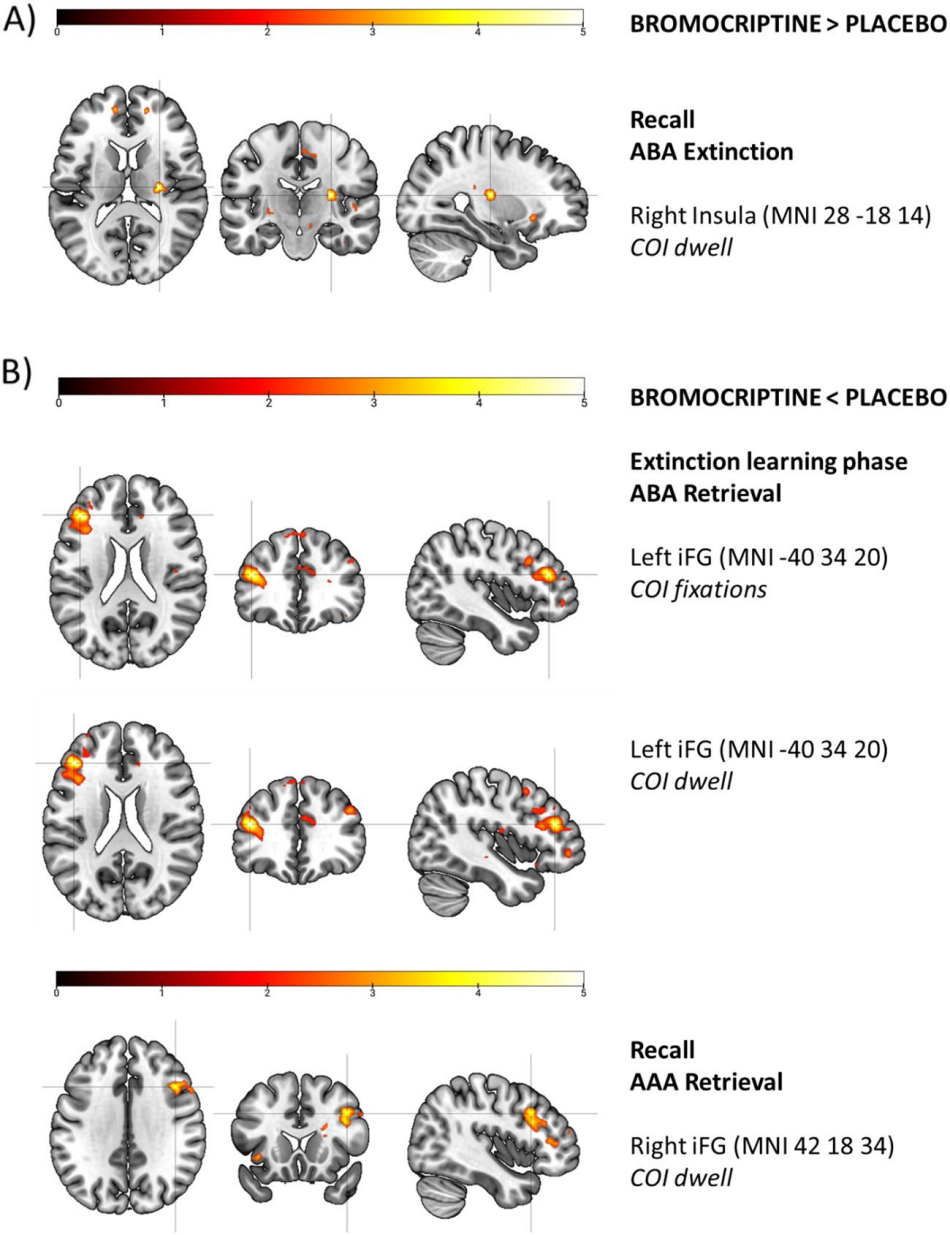
BOLD activation of extinction-related brain areas. (**A**) The bromocriptine group had greater BOLD activation, which correlated with dwell of contextual cues, in the right insula during recall of ABA extinction stimuli compared to placebo (**B**) Bromocriptine showed lower activation during retrieval conditions in extinction learning phase and recall. During the ABA retrieval condition in the extinction learning phase, BOLD activation in left iFG correlated with fixations and dwell of contextual cues and was lower in the bromocriptine group compared to placebo. Right iFG activation patterns correlated with dwell in right iFG during recall of AAA retrieval stimuli and were also lower in the bromocriptine group compared to placebo.

Compared to placebo, the bromocriptine group showed reduced BOLD activation in left iFG, which correlated with fixations and dwell, during the ABA retrieval condition of the extinction learning phase (see Fig. 5). During recall of AAA retrieval stimuli, the bromocriptine group had reduced activation in right iFG compared to placebo, which correlated with dwell. In contrast, greater BOLD activation patterns for the bromocriptine groupwere found exclusively in extinction conditions. In the AAA extinction condition, greater activation was observed in the right superior frontal gyrus (during the extinction learning phase) and paracentral lobule (during recall) and correlated with fixations. During the recall of ABA extinction stimuli, bromocriptine had activation-increasing effects in the right insula that correlated with dwell time (see Fig. 5).

Overall, fixations and dwell time on contextual information positively correlated with activation patterns in extinction-related brain areas of all participants (one-sample t-test) during extinction learning and recall irrespective of the treatment group. During the extinction learning phase, we revealed a correlation between fixation and activation of left iFG during ABA extinction. In the recall phase, fixations correlated with activation in the left parahippocampal gyrus during AAA extinction, and in the right insula during ABA retrieval. Also, during recall of ABA retrieval stimuli BOLD activation of the right hippocampus correlated with dwell time. See Table 4 for an overview of our imaging analysis results and Fig. 5 for differences in BOLD activation between treatment groups during task-relevant conditions. A negative correlation was observed between BOLD activation of the left anterior cingulate cortex and dwell during extinction learning of AAA retrieval stimuli.

**Table 4:** Overview imaging analysis.

	Area	BA	HEM	MNI	Voxel	*t*	*p*	*COI*
Bromocriptine > Placebo. Contrasts between treatment groups show higher activation in bromocriptine compared to the placebo group in extinction-related brain areas during acquisition, extinction, and recall of extinction memory. [Two-sample test, FWE-corrected p < 0.05, k = 10, on cluster level]								
**Extinction**								
AAA Extinction	Superior frontal gyrus	9/10	R	16 52 30	227	4.10	0.04	Fixations
**Recall**								
AAA Extinction	Paracentral Lobule	6	R	28 6 − 12	129	4.23	0.05	Fixations
ABA Extinction	Insula	13	R	28 − 18 14	92	4.95	0.01	Dwell
Bromocriptine < Placebo. Contrasts between treatment groups show higher activation in placebo compared to the bromocriptine group in extinction-related brain areas during acquisition, extinction, and recall of extinction memory. [Two-sample test, FWE-corrected p < 0.05, k = 10, on cluster level]								
**Extinction**								
ABA Retrieval	Inferior Frontal Gyrus	46	L	− 40 34 20	104	5.84	0.001	Fixations
	Inferior Frontal Gyrus	46	L	− 40 34 20	117	6.20	< 0.001	Dwell
**Recall**								
AAA Retrieval	Inferior Frontal Gyrus	45/46	R	42 18 34	51	4.19	0.012	Dwell
Overview of a positive correlation between BOLD activation of all participants and learning errors, fixations, and dwell as a covariate of interest (COI) [One-sample test, FWE-corrected p < 0.05, k = 10, on cluster level; *SVC peak level]								
**Extinction**								
ABA Extinction	Inferior Frontal Gyrus	47	L	− 28 22 18	108	5.12	0.024	Fixations
** Recall**								
AAA Extinction	Parahippocampal Gyrus*	28	L	− 20 −12 −24	65	4.46	0.024	Fixations
ABA Retrieval	Insula	13	R	− 34 − 26 10	118	5.04	0.01	Fixations
	Hippocampus	22	R	35 − 12 − 4	124	5.91	0.00	Dwell
Negative correlation between BOLD activation of all participants and dwell as a covariate of interest (COI) [One-sample test, FWE-corrected p < 0.05, k = 10, on cluster level]								
**Extinction**								
AAA Retrieval	Anterior Cingulate Cortex	32	L	− 14 42 6	116	4.83	0.05	Dwell

### Correlations of task performance and attention to contextual cues

During acquisition, we found a positive correlation between learning errors and fixations (r = 0.31, *p* = 0.03) and dwell time (r = 0.37, *p* = 0.01). Participants who had fewer fixations and less dwell on contextual cues showed lower error rates during the initial learning of associations.

**Figure 6:**
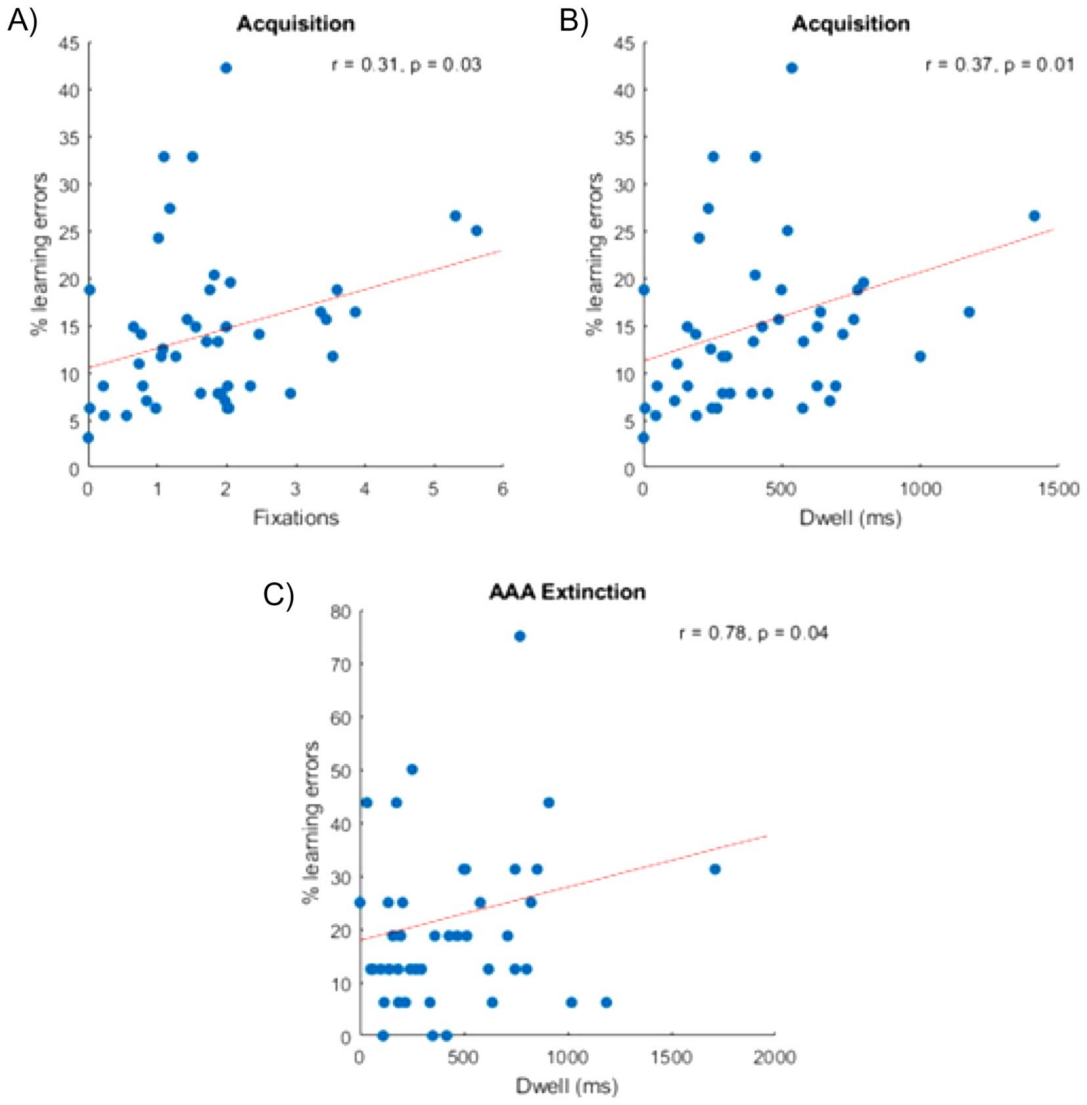
Positive correlation of task performance and fixation/dwell on contextual cues in task-relevant conditions. During the acquisition, we observed a positive correlation between learning errors and (**A**) fixations, and (**B**) dwell time. Participants with fewer acquisition errors had fewer fixations and less dwell on contextual cues. (**C**) In the extinction learning phase, participants who had a good extinction learning performance (i.e., fewer learning errors) had less dwell on contextual cues.

During the extinction learning phase, we revealed a positive correlation between learning errors and dwell on contextual cues for all participants in ABA retrieval (r = 0.72, *p* = 0.05) and AAA extinction conditions (r = 0.78, *p* = 0.04). In the recall phase, during ABA retrieval, we found a positive correlation between learning errors and fixations (r = 0.38, *p* = 0.008) and between learning errors and dwell (r = 0.33, *p* = 0.02). All participants had more recall errors for retrieval stimuli in the conditions where the context changed throughout the task phases (ABA) when they had more fixations and longer dwell on contextual cues. We observed no differences between both treatment groups in correlations of task performance and attention to contextual cues (in terms of fixations and dwell) during all task phases (for all correlations, we used Spearman's rank correlation; see Fig. 6 for a selective overview of significant correlations).

## Discussion

### Behavioural performance

#### Learning and memory performance during the predictive learning task

All participants had more fixations and dwell on food stimuli compared to contextual cues. This was likely due to the task instructions, that called participants’ attention to the food stimulus. In contrast, the context provided additional information that was helpful but not essential for solving the task. During the acquisition, participants learned stimulus outcome associations—no context change, or consequence change was introduced during this phase. Our results indicated that participants with more fixations and longer dwell time on contextual cues made more learning errors. A potential reason for this is that the additional context information, which—in contrast to the association between food stimuli and consequence—was unnecessary for task solving, may have been distracting, with such a distraction reflected in more learning errors.

During the extinction learning phase, the extinction condition is characterized by a change in the contingency between food stimuli and outcome, while in the retrieval condition, the contingency valid during acquisition remains the same. Here, the performance of the treatment groups differed: the placebo group had significantly higher error rates for extinction stimuli compared to retrieval for both ABA and AAA trial types, i.e., with and without context change. The bromocriptine group showed a comparable learning performance across all experimental conditions, but higher errors for the AAA retrieval condition, where neither the consequence nor the context changes. Thus, this group did not benefit from the fact that retrieval trials need no updating of outcome information, which usually should result in significantly lower error rates compared to extinction trials.

During ABA retrieval and AAA extinction conditions, fixation and dwell on contextual cues correlated with learning performance for all participants. In both conditions, higher error rates were associated with more fixations and dwell on context information. In the extinction learning phase, a change in the contingency between stimulus and outcome is introduced. To respond correctly, participants must adjust their behaviour. In ABA extinction, for the first time, contextual information may constitute an important mediator that supports successful extinction learning. Therefore, it may be helpful to incorporate contextual information during extinction learning. In contrast, in ABA retrieval and AAA extinction conditions, contextual cues do not provide relevant or novel information about a change in consequence. Presumably, more fixations and dwell on contextual cues in these trial types may lead to higher error rates since attention to irrelevant information may cause distraction.

During the recall of ABA extinction stimuli, the condition that is designed to evoke renewal, we observed no renewal. In other words, all participants successfully extinguished and retrieved stimulus outcome associations in conditions with and without context change. During our renewal test, the food stimulus was presented in the context of acquisition again. Participants recalled the stimulus outcome association they had previously learned during the extinction learning phase and ignored the reappearance of the stimulus in its initial context as well as the outcome that was linked to this context. For the bromocriptine group, we observed significantly more fixations on contextual cues during the recall of ABA extinction memory. However, the attention to the context was not reflected in recall performance (i.e., ABA renewal). We assume that contrary to our expectations, the modification of the task design in terms of more contextual information supported a better recall of extinction memory and therefore diminished renewal responses, compared to the task design that was used in previous studies.

#### Behavioural performance during our predictive learning task described in models of attention in associative learning

Our modified task design, containing more complex contextual information and more context cues, did not result in particularly high attention to contextual information per se. Even though the context provided essential information that may support learning and recall of stimulus-outcome associations, it was possible to solve the task without shifting attention to contextual cues in the acquisition and extinction learning phase. During acquisition, food stimuli were the best predictors for the correct behavioural response and presumably, therefore less attention was paid to contextual cues. In line with the attentional model of high predictiveness, participants shifted their attention toward food stimuli and neglected context. However, during the extinction learning and recall phase, contextual cues provided task-relevant information that may have supported encoding of the changed stimulus-outcome association and adjusting the response. During the extinction learning phase, individuals experienced a high level of uncertainty due to surprising changes in context (ABA trials) and stimulus-outcome associations in extinction trials. During ABA trials and extinction trials, we observed that participants who made more learning errors also shifted their attention to contextual cues—as indicated by more fixations and longer dwell. Here, our findings reflect the high uncertainty model of attention which proposes that individuals shift their attention to cues when they are uncertain about their consequences.

In addition, we observed the effects of dopaminergic receptor stimulation on attentional processes. During recall of ABA extinction stimuli, the bromocriptine group showed significantly more fixations on contextual cues as well as higher activation of the right insula that correlated with dwell compared to placebo. During this condition, participants experienced the highest uncertainty compared to other conditions during our predictive learning task. This finding supports the idea that the shift of attention towards contextual cues during recall of the high uncertainty condition (ABA extinction), results in a good recall of ABA extinction memory indicated by lack of renewal, which was presumably supported by dopaminergic D2-like receptor stimulation and activation of the right insula.

### Imaging analysis

#### BOLD activation of iFG during extinction learning and recall phases

Multiple neuroimaging studies demonstrated a contribution of iFG in the processing and retrieval of semantic information^43–45^ as well as in response inhibition and mediating conflicting response options^46^. The right iFG is considered to have a key role in executive control mechanisms such as response inhibition^47^. Previous studies have shown that neural activation in the right iFG increased during inhibitory control compared to baseline^48–50^. Along these lines, the right iFG functions as an inhibitory control mechanism that suppresses irrelevant responses^48,51^. It is assumed that the right iFG is functionally involved in the detection of cues that are relevant for goal-directed behaviour^52^. In our experiment, BOLD activation in the right iFG was higher when participants had longer dwell on contextual cues during AAA retrieval trials in the recall phase. In this condition, the food stimulus is always presented in the same context and no consequence change is introduced. Therefore, there is no need for response adaptation or inhibition of the previously learned motor response, instead a control mechanism may be activated since information derived from dwell on contextual cues tells whether a change in context or consequence requires adjustment of the response. The correlation with greater BOLD activation and dwell on contextual cues is in line with previous studies which demonstrate that the right iFG is functionally involved in the detection of cues that are relevant for goal-directed behaviour^52^.

The left iFG is also involved in executive functions and is assumed to play a key role in inhibitory control, the selection of semantic information^53^, and directly interferes with working memory^47,54^. Furthermore, it modulates the suppression of inappropriate responses^47^ and was also repeatedly found active during extinction learning where it is assumed to mediate the renewal effect and processing of conflicting response options during extinction^7,8,32,55^.

In our experiment, activation of the left iFG was greater during ABA conditions (retrieval and extinction) in the extinction learning phase. During the ABA extinction condition, inhibitory control mechanisms are required since the change of context indicates a change in contingency between the stimulus and the consequence which must be reflected in an adapted response. In this condition, the correlation of left iFG activation with fixations suggests that more fixations on contextual cues, which point towards higher attention to context information, resulted in greater activation of left iFG accompanied by a change of goal-directed behaviour. We observed a difference between treatment groups during this condition and a potential effect of dopamine D2-like receptors on iFG activation. Left iFG activation that correlated with dwell and fixations was reduced in the bromocriptine group compared to placebo. This finding is in line with previous studies that propose an influence of D2-like receptor availability on iFG activation and inhibitory response mechanisms^27,28^.

#### Processing of contextual information correlates with BOLD activation of the insula during conditions with context change (ABA)

We observed different activation patterns between treatment groups during the recall of ABA extinction stimuli. The insula is assumed to be functionally involved in the detection of salient sensory information and mediates cognitive control to guide attention and goal-directed behaviour^56–60^*.* The anterior insula is also part of a salience network together with the dorsal anterior cingulate cortex (ACC), which is activated and recruited during the detection and integration of salient external stimuli^60–62^. Dopamine D2-like receptors influence neural activation in the insula and therefore the dopaminergic system is assumed to mediate response inhibition^28^.

Our findings suggest a robust functional engagement of the right insula during recall of stimuli in conditions with context change, as this brain area was recruited during recall of stimulus-outcome associations after stimuli were presented in a different context. Also, we observed a potential effect of dopamine D2-like receptor stimulation on activation of the right insula that modulated attention and integration of salient contextual information during the most prominent condition of our task, i.e., recall of ABA extinction stimuli. During this condition, the bromocriptine group increased their attention to contextual cues, and we observed higher activation in the right insula that correlated with our measure of attention compared to placebo. Therefore, we can assume that the right insula was functionally involved during the detection of task-relevant contextual information.

#### Negative correlation of ACC activity with dwell during AAA Retrieval

The anterior cingulate cortex is a part of the salience network that responds to salient stimuli^63^. Many previous studies have proposed that the ACC is essential for executive control of cognition in terms of conflict monitoring^64,65^ and error detection^66–68^. In conflict monitoring theories, the ACC is assumed to detect upcoming conflicts by processing unpredictable sensory input during goal-directed attention^65,69^. The detection of these conflicts is then followed by changes in attention and information processing to adjust goal-directed behaviour. It is assumed that the ACC also plays a crucial role during object identification and reconsolidation of object memory^70,71^. In humans, the most pronounced activation in ACC was observed during tasks in which incongruent or competitive stimuli were presented and conflicts occurred. Our findings are in line with the literature that describes the ACC as a regulator of attention processes to adjust behaviour during conflict monitoring. Participants with a longer dwell on contextual cues showed reduced activation in the ACC during the AAA retrieval condition. Here, a longer dwell on contextual cues may have improved conflict monitoring which was accompanied by a reduced activation of ACC.

#### Medial temporal lobe activation during the recall phase

The hippocampus is considered essential for context-dependent recall of memories and processing context information^72,73^. Previous studies observed higher activation in HC during extinction learning^5^ and demonstrated functional involvement in the processing of context information^40,41,72^ as well as during recall of context-dependent memory^74^*.* In particular, the right HC is recruited during learning, extinction and recall of stimulus-outcome associations^4,5,73,75,76^. During our experiment, the activation of right HC was greater while we observed a longer dwell on contextual cues during ABA retrieval in the recall phase. These results are in line with previous findings which show that the right HC is functionally involved in the recall of stimulus-outcome associations as well as context information.

During recall of AAA extinction stimuli, the activation of the parahippocampal gyrus (PHC) correlated with fixations on contextual cues. The PHC is assumed to play a critical role during memory encoding and retrieval. Also, it is associated with multiple cognitive processes and is specially recruited during tasks where the processing of visuospatial information, spatial navigation, remembering locations of objects, and types of associative memory are relevant^77–79^. Participants who showed more fixations on contextual information had a more prominent activation in left PHC. These results are in line with previous findings that the PHC is functionally involved in the processing of visuospatial information during the recall of extinction memory.

### Limitations and future directions

One limitation of our study is our homogeneous group of participants, consisting exclusively of university students which do not represent the entire spectrum of interindividual differences in our society regarding their cognitive performance. Also, to further investigate the effects of D2-like receptor stimulation, a study design is required that examines intraindividual effects of dopaminergic modulation on attention and extinction learning.

Nevertheless, our results support the assumption that the dopaminergic system is crucial for learning and memory processes and has a particular role during the recall of context-dependent extinction memory. Based on our findings, we want to highlight the role of dopamine in the modulation of BOLD activity in extinction-related brain areas, which may influence attention to contextual cues and thus performance in extinction learning.

In a clinical context, a holistic understanding of the dopaminergic system in context-related extinction memory consolidation is of great interest to improve psychotherapy^80^. It has already been shown that the administration of L-Dopa after successful fear extinction results in improved extinction memory retrieval^81^. To effectively influence extinction-based therapy with dopaminergic stimulation and specifically to improve context-dependent extinction memory consolidation, to prevent renewal, it is important to understand the dopaminergic effects on cognitive functions and activity of extinction-related brain areas.

### Conclusion

In conclusion, contrary to our expectations, dopaminergic receptor activation as well as enhanced complexity of contextual information did not result in (a) higher attention in terms of more fixations and longer dwell on contextual cues compared to food stimuli, and (b) higher renewal rates in our predictive learning task. Nevertheless, during the context-dependent recall of extinction memory, the bromocriptine group showed significantly higher attention to contextual cues compared to placebo. The modified task design with its many contextual cues probably abolished renewal, since renewal had been present in previous studies using the old task design. Therefore, an exposure-based therapy might benefit from a combination of D2-like receptor stimulation and a multitude of contextual cues to prevent context-dependent failure of extinction memory. During recall of ABA extinction memory, dopaminergic receptor stimulation had however effects on attention to contextual information in terms of more fixations for the bromocriptine group compared to placebo. These differences in attentional processing occurred during the task condition where context information was most prominent. The increase in complexity of contextual information in our modified task resulted in an almost complete lack of renewal responses in both treatment groups. Taken together, we assume that dopaminergic receptor stimulation modulates attention to contextual cues only in a condition where contextual information is most prominent in our task. In addition, dopamine affected BOLD activation in extinction-related brain areas during extinction learning and recall. BOLD activation correlated with our measure of attention on contextual information and was lower in extinction-related brain areas during retrieval conditions as well as greater during extinction conditions, compared to placebo.

## Data Availability

Raw data was generated at the University Hospital Bergmannsheil Bochum. Behavioural, eye-tracking, and imaging data, as well as the code we used for data analysis, can be made available upon reasonable request with the need for a formal data-sharing agreement. Please contact the corresponding author Alina Nostadt (alina.nostadt@rub.de) regarding data.

## References

[1] Myers KM, Davis M (2007). Mechanisms of fear extinction. Mol. Psychiatry.

[2] Bouton ME, Bolles RC (1979). Contextual control of the extinction of conditioned fear. Learn. Motiv..

[3] Lucke S, Lachnit H, Stüttgen MC, Uengoer M (2014). The impact of context relevance during extinction learning. Learn. Behav..

[4] Kinner VL, Merz CJ, Lissek S, Wolf OT (2016). Cortisol disrupts the neural correlates of extinction recall. Neuroimage.

[5] Lissek S, Glaubitz B, Uengoer M, Tegenthoff M (2013). Hippocampal activation during extinction learning predicts occurrence of the renewal effect in extinction recall. Neuroimage.

[6] Lissek S, Glaubitz B, Wolf OT, Tegenthoff M (2015). The DA antagonist tiapride impairs context-related extinction learning in a novel context without affecting renewal. Front. Behav. Neurosci..

[7] Lissek S, Golisch A, Glaubitz B, Tegenthoff M (2017). The GABAergic system in prefrontal cortex and hippocampus modulates context-related extinction learning and renewal in humans. Brain Imaging Behav..

[8] Lissek S, Klass A, Tegenthoff M (2020). Left inferior frontal gyrus participates in mediating the renewal effect irrespective of context salience. Front. Behav. Neurosci..

[9] Marchetti G (2014). Attention and working memory: Two basic mechanisms for constructing temporal experiences. Front. Psychol..

[10] Kane MJ, Bleckley MK, Conway ARA, Engle RW (2001). A controlled-attention view of working-memory capacity. J. Exp. Psychol. Gen..

[11] Pearce, J. Mackintosh, N. Two theories of attention: A review and a possible integration (2010).

[12] Pearce JM, Hall G (1980). A model for Pavlovian learning: Variations in the effectiveness of conditioned but not of unconditioned stimuli. Psychol. Rev..

[13] George DN, Pearce JM (2012). A configural theory of attention and associative learning. Learn. Behav..

[14] Darby RJ, Pearce JM (1995). Effects of context on responding during a compound stimulus. J. Exp. Psychol. Anim. Behav. Process..

[15] Mackintosh NJ (1975). A theory of attention: Variations in the associability of stimuli with reinforcement. Psychol. Rev..

[16] Esber GR, Haselgrove M (2011). Reconciling the influence of predictiveness and uncertainty on stimulus salience: A model of attention in associative learning. Proc. R. Soc. B Biol. Sci..

[17] Nasser HM, Calu DJ, Schoenbaum G, Sharpe MJ (2017). The dopamine prediction error: Contributions to associative models of reward learning. Front. Psychol..

[18] El-Ghundi M, O’Dowd BF, George SR (2007). Insights into the role of dopamine receptor systems in learning and memory. Rev. Neurosci..

[19] Lissek S, Glaubitz B, Klass A, Tegenthoff M (2018). The effects of dopaminergic D2-like receptor stimulation upon behavioral and neural correlates of renewal depend on individual context processing propensities. Neuroimage.

[20] Camps M, Kelly PH, Palacios JM (1990). Autoradiographic localization of dopamine D1 and D2 receptors in the brain of several mammalian species. J. Neural. Transm..

[21] Hurd YL, Suzuki M, Sedvall GC (2001). D1 and D2 dopamine receptor mRNA expression in whole hemisphere sections of the human brain. J. Chem. Neuroanat..

[22] Khan ZU (2000). Dopamine D5 receptors of rat and human brain. Neuroscience.

[23] Meador-Woodruff JH (1996). Dopamine receptor mRNA expression in human striatum and neocortex. Neuropsychopharmacology.

[24] Vincent SL, Khan Y, Benes FM (1995). Cellular colocalization of dopamine D1 and D2 receptors in rat medial prefrontal cortex. Synapse.

[25] McNamara CG, Tejero-Cantero Á, Trouche S, Campo-Urriza N, Dupret D (2014). Dopaminergic neurons promote hippocampal reactivation and spatial memory persistence. Nat. Neurosci..

[26] Gerlicher AMV, Tüscher O, Kalisch R (2018). Dopamine-dependent prefrontal reactivations explain long-term benefit of fear extinction. Nat. Commun..

[27] Ghahremani DG (2012). Striatal dopamine D2/D3 receptors mediate response inhibition and related activity in frontostriatal neural circuitry in humans. J. Neurosci..

[28] Pfeifer P (2022). Prefrontal and striatal dopamine D2/D3 receptors correlate with fMRI BOLD activation during stopping. Brain Imaging Behav..

[29] Nieoullon A (2002). Dopamine and the regulation of cognition and attention. Prog. Neurobiol..

[30] Üngör M, Lachnit H (2006). Contextual control in discrimination reversal learning. J. Exp. Psychol. Anim. Behav. Process..

[31] Üngör M, Lachnit H (2008). Dissociations among ABA, ABC, and AAB recovery effects. Learn. Motiv..

[32] Lissek S, Klass A, Tegenthoff M (2019). Effects of noradrenergic stimulation upon context-related extinction learning performance and BOLD activation in hippocampus and prefrontal cortex differ between participants showing and not showing renewal. Front. Behav. Neurosci..

[33] Lissek S, Glaubitz B, Güntürkün O, Tegenthofl M (2015). Noradrenergic stimulation modulates activation of extinction-related brain regions and enhances contextual extinction learning without affecting renewal. Front. Behav. Neurosci..

[34] Lang S (2009). Context conditioning and extinction in humans: Differential contribution of the hippocampus, amygdala and prefrontal cortex. Eur. J. Neurosci..

[35] Lissek S, Glaubitz B, Schmidt-Wilcke T, Tegenthoff M (2016). Hippocampal context processing during acquisition of a predictive learning task is associated with renewal in extinction recall. J. Cognit. Neurosci..

[36] Mumford JA (2012). A power calculation guide for fMRI studies. Soc. Cognit. Affect. Neurosci..

[37] Faul F, Erdfelder E, Lang AG, Buchner A (2007). G*Power 3: A flexible statistical power analysis program for the social, behavioral, and biomedical sciences. Behav. Res. Methods.

[38] Holt RIG, Barnett AH, Bailey CJ (2010). Bromocriptine: Old drug, new formulation and new indication. Diabetes Obes. Metab..

[39] Klass A, Glaubitz B, Tegenthoff M, Lissek S (2017). d-Cycloserine facilitates extinction learning and enhances extinction-related brain activation. Neurobiol. Learn. Mem..

[40] Kalisch R (2006). Context-dependent human extinction memory is mediated by a ventromedial prefrontal and hippocampal network. J. Neurosci..

[41] Milad MR (2007). Recall of fear extinction in humans activates the ventromedial prefrontal cortex and hippocampus in concert. Biol. Psychiatry.

[42] Tzourio-Mazoyer N (2002). Automated anatomical labeling of activations in SPM using a macroscopic anatomical parcellation of the MNI MRI single-subject brain. Neuroimage.

[43] Thompson-Schill SL, D’Esposito M, Aguirre GK, Farah MJ (1997). Role of left inferior prefrontal cortex in retrieval of semantic knowledge: A reevaluation. Proc. Natl. Acad. Sci..

[44] Buckner RL (1995). Functional anatomical studies of explicit and implicit memory retrieval tasks. J. Neurosci..

[45] Démonet JF (1992). The anatomy of phonological and semantic processing in normal subjects. Brain.

[46] Konishi S (1999). Contribution of working memory to transient activation in human inferior prefrontal cortex during performance of the Wisconsin card sorting test. Cereb. Cortex.

[47] Swick D, Ashley V, Turken AU (2008). Left inferior frontal gyrus is critical for response inhibition. BMC Neurosci..

[48] Aron AR, Robbins TW, Poldrack RA (2004). Inhibition and the right inferior frontal cortex. Trends Cognit. Sci..

[49] Rubia K, Smith AB, Brammer MJ, Taylor E (2003). Right inferior prefrontal cortex mediates response inhibition while mesial prefrontal cortex is responsible for error detection. Neuroimage.

[50] Menon V, Adleman NE, White CD, Glover GH, Reiss AL (2001). Error-related brain activation during a Go/NoGo response inhibition task. Hum. Brain Mapp..

[51] Van Boxtel GJM, Van der Molen MW, Jennings JR, Brunia CHM (2001). A psychophysiological analysis of inhibitory motor control in the stop-signal paradigm. Biol. Psychol..

[52] Hampshire A, Chamberlain SR, Monti MM, Duncan J, Owen AM (2010). The role of the right inferior frontal gyrus: Inhibition and attentional control. Neuroimage.

[53] Thompson-Schill SL (1998). Verb generation in patients with focal frontal lesions: A neuropsychological test of neuroimaging findings. Proc. Natl. Acad. Sci..

[54] Thompson-Schill SL (2002). Effects of frontal lobe damage on interference effects in working memory. Cogn. Affect. Behav. Neurosci..

[55] Bouton ME, Trask S, Carranza-Jasso R (2016). Learning to inhibit the response during instrumental (operant) extinction. J. Exp. Psychol. Anim. Learn. Cogn..

[56] Craig AD (2009). How do you feel—Now? The anterior insula and human awareness. Nat. Rev. Neurosci..

[57] Uddin LQ (2015). Salience processing and insular cortical function and dysfunction. Nat. Rev. Neurosci..

[58] Menon V, Uddin LQ (2010). Saliency, switching, attention and control: A network model of insula function. Brain Struct. Funct..

[59] Nieuwenhuys, R. The insular cortex. A review. In *Progress in Brain Research* Vol. 195 123–163 (Elsevier B.V., 2012). 10.1016/B978-0-444-53860-4.00007-6 22230626

[60] Cai W, Ryali S, Chen T, Li CSR, Menon V (2014). Dissociable roles of right inferior frontal cortex and anterior insula in inhibitory control: Evidence from intrinsic and task-related functional parcellation, connectivity, and response profile analyses across multiple datasets. J. Neurosci..

[61] Swick D, Ashley V, Turken U (2011). Are the neural correlates of stopping and not going identical? Quantitative meta-analysis of two response inhibition tasks. Neuroimage.

[62] Levy BJ, Wagner AD (2011). Cognitive control and right ventrolateral prefrontal cortex: Reflexive reorienting, motor inhibition, and action updating. Ann. N. Y. Acad. Sci..

[63] Qadir H (2018). Structural connectivity of the anterior cingulate cortex, claustrum, and the anterior insula of the mouse. Front. Neuroanat..

[64] Ebitz RB, Platt ML (2015). Neuronal activity in primate dorsal anterior cingulate cortex signals task conflict and predicts adjustments in pupil-linked arousal. Neuron.

[65] Botvinick MM, Cohen JD, Carter CS (2004). Conflict monitoring and anterior cingulate cortex: An update. Trends Cognit. Sci..

[66] Brown JW, Braver TS (2005). Learned predictions of error likelihood in the anterior cingulate cortex. Science.

[67] Ito S, Stuphorn V, Brown JW, Schall JD (2003). Performance monitoring by the anterior cingulate cortex during saccade countermanding. Science.

[68] Wu D (2017). Persistent neuronal activity in anterior cingulate cortex correlates with sustained attention in rats regardless of sensory modality. Sci. Rep..

[69] Dignath D, Eder AB, Steinhauser M, Kiesel A (2020). Conflict monitoring and the affective-signaling hypothesis—An integrative review. Psychon. Bull. Rev..

[70] Weible AP, Rowland DC, Pang R, Kentros C (2009). Neural correlates of novel object and novel location recognition behavior in the mouse anterior cingulate cortex. J. Neurophysiol..

[71] Weible AP, Rowland DC, Monaghan CK, Wolfgang NT, Kentros CG (2012). Neural correlates of long-term object memory in the mouse anterior cingulate cortex. J. Neurosci..

[72] Smith DM, Mizumori SJY (2006). Learning-related development of context-specific neuronal responses to places and events: The hippocampal role in context processing. J. Neurosci..

[73] Maren S (2011). Seeking a spotless mind: Extinction, deconsolidation, and erasure of fear memory. Neuron.

[74] Kennedy PJ, Shapiro ML (2004). Retrieving memories via internal context requires the hippocampus. J. Neurosci..

[75] Orsini CA, Kim JH, Knapska E, Maren S (2011). Hippocampal and prefrontal projections to the basal amygdala mediate contextual regulation of fear after extinction. J. Neurosci..

[76] Milad MR, Quirk GJ (2012). Fear extinction as a model for translational neuroscience: Ten years of progress. Annu. Rev. Psychol..

[77] Aminoff EM, Kveraga K, Bar M (2013). The role of the parahippocampal cortex in cognition. Trends Cognit. Sci..

[78] Aguirre GK, Detre JA, Alsop DC, D’Esposito M (1996). The parahippocampus subserves topographical learning in man. Cereb. Cortex.

[79] Epstein R, Kanwisher N (1998). A cortical representation the local visual environment. Nature.

[80] Haaker J (2013). Single dose of l-dopa makes extinction memories context-independent and prevents the return of fear. Proc. Natl. Acad. Sci..

[81] Gerlicher AMV, Tüscher O, Kalisch R (2019). L-DOPA improves extinction memory retrieval after successful fear extinction. Psychopharmacology.

